# Influence of High-Genetic-Potential Lineages and Sex on Carcass Traits and Pork Quality

**DOI:** 10.3390/ani16081186

**Published:** 2026-04-14

**Authors:** Érika Nayara Freire Cavalcanti, Daniel Rodrigues Dutra, Erick Alonso Villegas-Cayllahua, Heloisa de Almeida Fidelis, Aline Giampietro-Ganeco, Mateus Roberto Pereira, Fábio Borba Ferrari, Romário Alves Rodrigues, Marco Antonio de Andrade Belo, Hirasilva Borba

**Affiliations:** 1 Faculty of Agricultural and Veterinary Sciences, São Paulo State University, Jaboticabal 14884-900, Brazil; danielrdutra@hotmail.com (D.R.D.); evillegasc22@gmail.com (E.A.V.-C.); heloisa.a.fidelis@gmail.com (H.d.A.F.); mateusscj2012@hotmail.com (M.R.P.); fbf_zoo@hotmail.com (F.B.F.); romario.a.rodrigues@unesp.br (R.A.R.); maabelo@hotmail.com (M.A.d.A.B.); hirasilva.borba@unesp.br (H.B.); 2 School of Animal Science and Food Engineering, University of São Paulo, Pirassununga 13635-900, Brazil; giampietroganeco@gmail.com

**Keywords:** chemical composition, genotype, longissimus, meat quality, sensory analysis, swine production

## Abstract

This study evaluated the effects of sex and genetics on carcass traits and pork quality in 180 carcasses from castrated males and females of three commercial lines: A (Pietrain × Duroc × Large White × Landrace), B (Line A × Pietrain) and C (Line A × Hampshire). Males showed greater backfat (14.05 vs. 13.38%) and lower lean meat (58.65 vs. 59.08%) than females. Line C exhibited the highest marbling score (2.82) and hot and cold carcass weights (90.04 and 88.05 kg), while Line B achieved the highest ham yield (32.5%). Castrated males had higher marbling (2.74 vs. 2.48) but lower shear force (3.68 vs. 3.90 kgf) than females. Line C showed the highest marbling (2.82) and shear force (4.04 kgf). Males of Line C had longer sarcomeres than females of the same line and males from Line B. Males exhibited higher protein (24.73 vs. 23.91%) but lower mineral content (1.55 vs. 1.85%) than females. Line A had the highest mineral content (1.90%). Collagen content was highest in males from Lines A and B (insoluble: 0.35–0.38%; soluble: 0.09%; total: 0.44–0.47%), and lower in Line C (insoluble: 0.18%; soluble: 0.04%; total: 0.21%). These results indicate that sex and new genetic lines influence carcass composition and instrumental meat quality traits.

## Simple Summary

This study explored how sex and genetic background influence carcass traits and pork quality in commercial pigs, providing insights with practical value for the swine industry and consumers. Castrated males and females from three high-performance genetic lines were analyzed for carcass composition, meat physicochemical traits and sensory quality. Results showed that sex and genetic line affect traits such as sarcomere length, collagen content, backfat thickness, marbling and protein levels. Despite these differences, sensory quality, such as tenderness, juiciness and flavor, remained stable across all groups. These results highlight how modern high-performance commercial lines express different carcass and muscle characteristics while still maintaining consistent eating quality. By revealing how contemporary genetic backgrounds interact with sex to shape these traits, the study offers updated evidence that supports informed decision-making in breeding and production systems. By understanding these influences, producers can make informed decisions to improve efficiency, maximize carcass value and better meet consumer preferences. Moreover, maintaining consistent sensory quality supports consumer satisfaction and market acceptance, ultimately contributing to a more competitive and sustainable pork industry.

## 1. Introduction

One of the main concerns in pork production is how to improve carcass traits and meat quality to meet consumer market demands. It is well established that various factors influence carcass and meat quality [1], among which genotype is one of the primary determinants of carcass composition and overall value in pigs [2]. The swine industry has adopted specific commercial genetic lines to take advantage of heterosis effects on key economic traits, particularly in producing carcasses with high muscle yield. It is well recognized that different breeds and sexes exhibit distinct characteristics in terms of carcass composition and meat quality [3,4].

Commercial pigs exhibit high muscle development, which raises concerns about potential negative impacts on meat quality [5]. It is known that muscularity and meat quality traits tend to be negatively correlated in genetic improvement programs [6,7]. The selection of genotypes for increased muscle yield, higher growth rate, and improved conformation has resulted in adverse effects on meat quality, including reduced intramuscular fat, a greater proportion of glycolytic muscle fibers, and increased occurrence of pale, soft, and exudative (PSE) meat [8,9]. Sex also plays a key role in determining carcass traits and meat quality [2]. It is known that entire males are more efficient in protein deposition than females, followed by castrated males [10,11]. Sex also affects both sensory and technological meat quality parameters [12].

The concept of meat quality is complex and encompasses physicochemical and sensory attributes that determine the final quality of the product. Although numerous studies have assessed pork quality using both instrumental and sensory approaches, instrumental analyses still predominate in the literature, and fewer studies integrate carcass traits, detailed physicochemical measurements, and sensory evaluation within the same experimental framework [6,13,14]. Consumer acceptance and purchase intent are strongly influenced by perceived quality attributes [15]. The primary goal of improving pork quality is to enhance the product’s value while satisfying consumer expectations for fresh and/or processed pork. In this context, questions remain as to what extent sex and commercial genetic lines—selected for high muscle yield, rapid growth, and superior conformation—affect pork quality and its sensory acceptance by consumers. Thus, the objective of this study was to evaluate the influence of sex and genetic line on carcass traits and the physicochemical and sensory quality of pork.

## 2. Materials and Methods

### 2.1. Location, Carcass Traits and Sample Collection

This study was conducted at the Laboratory of Animal-Origin Food Analysis (LaOra), Faculty of Agricultural and Veterinary Sciences (FCAV), São Paulo State University (UNESP), Jaboticabal campus, São Paulo, Brazil (21°08′ S, 48°11′ W, 583 m altitude), and it was approved by the Ethics Committee on the Use of Animals (CEUA) of the institution (protocol n. 2907/21, approved in 28 October 2021).

A total of 180 pig carcasses (90 surgically castrated males and 90 females) were evaluated, with each genetic line consisting of 60 pigs (30 males and 30 females). All males were surgically castrated at 3 days of age. Animals were born in the same season and raised on a single farm in Minas Gerais, Brazil, under identical management and feeding conditions, ensuring uniformity in environmental and nutritional factors. The pigs were slaughtered at 168 days of age, with an average live weight of 125 kg. Animals belonged to three commercial genetic lines: Line A (a synthetic purebred line composed of Pietrain, Duroc, Large White, and Landrace, selected for maximum profitability, growth efficiency, and carcass value); Line B (a hybrid of Line A × Pietrain, combining carcass conformation, growth efficiency, and robustness); and Line C (a hybrid of Line A × Hampshire, characterized by high profitability, growth performance, and robustness). Slaughter was carried out at a commercial abattoir in the state of Minas Gerais, Brazil, under official inspection by the Federal Inspection Service.

Carcass grading was performed on the slaughter line using an electronic grading probe (HENNESSY Grading Systems GP4/BP4, Hennessy Grading Systems Ltd., Auckland, New Zealand). The probe was inserted into the left half-carcass between the last thoracic and first lumbar vertebrae. The optical sensor at the tip of the probe measured backfat thickness (BT, mm), muscle depth (MD, mm), and lean meat percentage (LMP, %). Hot carcass weight (HCW, kg) was also recorded at this stage.

After 24 h of chilling at 0–2 °C, carcasses were weighed again to determine cold carcass weight (CCW, kg), then cut, deboned, and separated into commercial cuts, following Brazilian pork processing specifications [16]. The yield of loin, ham, rack and shoulder was calculated as a percentage of each cut in relation to cold carcass weight.

To standardize sampling and ensure anatomical representativeness, the entire loin corresponding to the Longissimus thoracis et lumborum muscle was removed from the right half-carcass of each animal 24 h post-slaughter. After removal, each loin was immediately vacuum-packed and subjected to rapid freezing in a blast freezer (−20 °C). This step enabled uniform and accelerated freezing, minimizing structural damage to the muscle tissue and preserving the physicochemical and sensory characteristics of the meat. The frozen samples were transported to the laboratory in a refrigerated vehicle, ensuring continuous maintenance of the cold chain. Carcasses were previously identified by sex and genetic line, with 30 replicates per treatment, totaling 180 samples for subsequent analyses.

### 2.2. Meat Quality Analyses

In the laboratory, each Longissimus thoracis et lumborum (LTL) muscle was thawed under controlled refrigeration (4 °C) for 24 h. Physicochemical analyses were performed on samples collected from the cranial portion of the LTL. All procedures were standardized to ensure reproducibility and to minimize variations associated with sample collection, transport, and preparation. pH was measured in duplicate in samples of the LTL muscle using a digital pH meter (Testo 205, Testo Inc., Sparta, NJ, USA) equipped with a penetration electrode, by direct insertion into the muscle. Color was determined using a Minolta Chrome Meter CR-400 colorimeter (Konica Minolta Sensing, Inc., Osaka, Japan) employing the CIELAB system (L*, a*, b*). Parameters evaluated included lightness (L*), redness (a*), and yellowness (b*) on 2.5 cm thick steaks cut from the LTL muscle. Measurements were taken at three different points on each sample to obtain an average value. Marbling score was assessed on 2.5 cm thick LTL steaks using the National Pork Producers Council (NPPC) marbling standards, with a scale from 1 (devoid) to 10 (abundant) [17]. Water-holding capacity (WHC) was determined by applying pressure to the muscle tissue. Two grams of LTL muscle were trimmed and finely chopped with a scalpel to obtain a uniform sample, placed between two filter papers and acrylic plates, then subjected to 10 kg of pressure for 5 min. The sample was subsequently weighed to calculate retained water as a percentage using the formula: (Final weight × 100)/Initial weight [18].

Cooking loss (CL) was determined following the methodology described in the Meat Cookery and Sensory Evaluation Manual [19]. Samples were cooked on a grill (George Foreman GBZ80, Spectrum Brands, Madison, WI, USA) until reaching an internal temperature of 71 °C, monitored in real-time with a thermocouple probe (FE-MUX, Flyever Indústria e Comércio de Equipamentos Eletrônicos Ltda., São Carlos, Brazil). Samples were weighed before and after cooking. Shear force (SF) was analyzed according to Wheeler et al. [20]. Cylindrical cores of 1.27 cm diameter were cut perpendicular to the muscle fiber orientation and subjected to shear using a Warner-Bratzler blade mounted on a texture analyzer (Texture Analyser TA-XT2i, Stable Micro Systems, Godalming, UK).

The sarcomere length was determined by phase contrast microscopy. Approximately 0.5 g of raw muscle was homogenized in a Turrax homogenizer (MA 102, Marconi Equipamentos de Laboratório Ltda, Piracicaba, Brazil) with 30 mL of a KCl (potassium chloride) 0.08 mol/L and KI (potassium iodide) 0.08 mol/L mixed solution (50:50) at over 15,000 rpm for 30 s to break cells and release myofibrils. A drop of the homogenate was placed on a microscope slide and covered with a coverslip. Observations were made using a phase contrast microscope (Novel BM2100, Nanjing Jlangnan Novel Optics., Ltd., Nanjing, China) at 1000× magnification (100× objective, 10× ocular).

Total, soluble and insoluble collagen concentrations were quantified by measuring hydroxyproline content following the method described by Cavalcanti et al. [21]. Five grams of frozen raw meat were weighed into 50 mL Falcon tubes and mixed with 20 mL of distilled water. Samples were incubated in a water bath at 80 °C for 2 h, then homogenized in an Ultra-Turrax (MA 102, Marconi Equipamentos de Laboratório Ltda) at 22,000 rpm for 1 min and centrifuged (HITACHI CR22N, Hitachi, Tokyo, Japan) at 4000 rpm for 15 min at room temperature (24 °C). The supernatant and sediment were separated and transferred to autoclavable tubes. Thirty milliliters of 6N HCl was added to the supernatant and 50 mL to the sediment. Samples were hydrolyzed in an autoclave (Phoenix AV-75Plus, Araraquara, Brazil) at 120 °C and 1 atm for 4 h. The following day, the pH of all samples was adjusted to 6.0 using 2N NaOH. Samples were then filtered into volumetric flasks (sediment in 250 mL flasks, supernatant in 100 mL flasks) and topped with distilled water. Aliquots of 10 mL were pipetted in duplicate into test tubes, where 1 mL of oxidation reagent (1.41% Chloramine-T) and 1 mL of color reagent (10 g p-dimethylaminobenzaldehyde in 35 mL 60% perchloric acid and 65 mL isopropanol) were added. Tubes were incubated in a water bath at 60 °C for 15 min. Absorbance was read using a spectrophotometer (Shimadzu UV-1800, Kyoto, Japan) at 560 nm. Soluble collagen concentration was determined from the supernatant samples, and insoluble collagen from sediment samples. A standard curve was prepared using hydroxyproline solutions of known concentration, with collagen concentration calculated as 7.14 times the hydroxyproline concentration. Collagen content (%) was calculated using the following equations:

- Insoluble collagen (%) = (Absorbance × F* × 250 × 100 × 7.14** × 10^−6^ × 100)/(10 × 2 × sample weight (g))
- Soluble collagen (%) = (Absorbance × F* × 100 × 50 × 7.14** × 10^−6^ × 100)/(10 × 2 × sample weight (g))
- Total collagen (%) = Soluble collagen (%) + Insoluble collagen (%)

where:

*F = 8.33, the mean absorbance value equivalent to 1 mg hydroxyproline from the standard curve prepared identically to the samples.

**7.14 is the conversion factor from hydroxyproline to collagen, assuming hydroxyproline represents 14% of collagen content.

Chemical composition analyses included moisture (method no. 950.46), protein (method no. 977.14), and ash (method no. 920.153) according to the Association of Official Analytical Chemists [22]. Fat content was determined using the Bligh and Dyer method [23].

### 2.3. Sensory Analysis

The sensory analysis was approved by the Human Research Ethics Committee through the Plataforma Brasil system—UNESP, Faculty of Agricultural and Veterinary Sciences, Jaboticabal campus (CAAE: 60634222.3.0000.9029; approval number 5.743.905; approved on 7 November 2022). A total of 114 untrained consumers were recruited on campus through printed posters displayed in common areas of the university. Recruitment occurred on a voluntary, walk-in basis, where individuals who passed by the sensory laboratory were invited to participate until the target sample size was reached. Eligibility criteria included being 18 years of age or older, being a regular pork consumer, and reporting no allergies or intolerances related to pork meat. The study included 114 consumers (55 females and 59 males), aged 18 to 61 years (mean age: 21 years). This sample size meets the recommended minimum for consumer acceptance tests (≥100 participants), allowing for reliable assessment of consumer preferences. Sensory evaluation was carried out in multiple sessions with six participants per session, conducted in individual booths under controlled lighting and temperature. Each session lasted approximately 20 min. Each evaluator expressed their opinion on the samples considering the attributes of color, flavor, odor, texture, and overall acceptance. A nine-point hedonic scale was used for the acceptance test, where panelists rated the samples as follows: 1—dislike extremely, 2—dislike very much, 3—dislike moderately, 4—dislike slightly, 5—neutral, 6—like slightly, 7—like moderately, 8—like very much, and 9—like extremely.

Standardized steaks were prepared from the cranial portion of the Longissimus thoracis et lumborum (LTL) muscle, collected from the right half-carcass and separated by sex and genetic line. This sampling approach ensured anatomical consistency and minimized variability due to intramuscular heterogeneity. Samples were prepared using a grill (George Foreman GBZ80), where steaks approximately 2.5 cm thick were cooked until reaching an internal temperature of 71 °C [19]. Each steak was then cut into six pieces. During each sensory panel session, three samples (one sex × three genetic lines) were served in randomized order and coded with three-digit numbers. Panelists cleansed their palates with crackers and water before tasting each sample. The acceptance test was conducted after verifying compliance with Brazilian microbiological standards for total coliforms and thermotolerant coliforms at 45 °C per gram (all samples showed counts < 3.0 × 10^0^), absence of *Salmonella* spp. in 25 g (all samples were *Salmonella* spp. negative), coagulase-positive *Staphylococcus* per gram (Line B females—1.0 × 10^3^; other samples < 1.0 × 10^2^), mesophilic bacteria per gram (all samples < 1.0 × 10^2^), and psychrotrophic bacteria per gram (Line A males—2.9 × 10^4^; Line A females—1.0 × 10^5^; Line C males—3.0 × 10^2^; other samples < 1.0 × 10^2^) [24]. Although some samples showed higher psychrotrophic counts, it is important to emphasize that Brazilian microbiological regulations for pork (RDC 331/2019 and IN 60/2019) do not establish limits for psychrotrophic bacteria in raw meat. Psychrotrophic microorganisms are primarily associated with shelf-life under prolonged refrigeration and are not used as safety criteria. All cuts included in the sensory analysis complied with the legally regulated parameters—absence of *Salmonella* spp., acceptable levels of thermotolerant coliforms, coagulase-positive *Staphylococcus*, and total mesophilic bacteria—and none of the samples showed any signs of sensory spoilage (off-odors, discoloration, or undesirable texture) during handling or preparation.

It is also important to note that the samples were vacuum-packed and frozen at −20 °C, then transported under refrigeration to the laboratory. Vacuum packaging and frozen storage substantially inhibit bacterial proliferation and delay the development of spoilage microorganisms, as extensively demonstrated in the literature. Under these conditions, psychrotrophic counts detected after thawing reflect surviving populations rather than active growth. In our procedures, all samples were thawed immediately prior to cooking and sensory evaluation, and no visual, textural, or odor deviations were reported by the panelists. Therefore, despite numerical differences in psychrotrophic counts, the storage conditions and timely preparation ensured that the meat was not deteriorated at the time of sensory testing, and these counts did not bias consumer acceptance results.

### 2.4. Statistical Analysis

A completely randomized design with a 3 × 2 factorial arrangement was adopted, consisting of three genetic groups (Lines A, B and C) and two sexes (surgically castrated males and females), with 30 replicates per treatment, totaling 180 samples. Each animal was treated as an independent experimental unit, with no hierarchical structure (e.g., pen, litter, sire, or slaughter batch) applicable to the dataset. Therefore, data were analyzed using the MIXED procedure of SAS 9.3 [25], considering fixed effects of sex, genetic group, and their interaction. Means were compared using Tukey’s test at a 5% significance level.

## 3. Results

Significant differences between sexes were observed for backfat thickness, lean meat percentage and ham yield (Table 1). Surgically castrated males showed higher backfat thickness and lower lean meat percentage and ham yield (14.05 mm, 58.65%, and 31.88%, respectively) compared to females (13.38 mm, 59.08%, and 32.29%, respectively).

No statistical interaction or difference between genetic groups and sex was found for muscle thickness or yields of loin, rack, and shoulder cuts. However, significant differences among genetic groups were observed for hot carcass weight, cold carcass weight, and ham yield. Line C showed the highest hot and cold carcass weights and the lowest ham yield (90.04 kg, 88.05 kg, and 31.64%, respectively) compared to lines A (88.60 kg, 86.05 kg, and 32.50%, respectively) and B (85.25 kg, 82.67 kg, and 32.50%, respectively). However, line A did not differ significantly from the other lines in carcass traits. Line A comprises crosses of lines B (line A × Pietrain) and C (line A × Hampshire), which likely explains the observed statistical similarity.

There was a significant interaction between sex and genetic group for sarcomere length (Table 2). Castrated males from line C showed greater sarcomere length values (1.99 µm) compared to males from line B and females from line C (1.94 µm and 1.92 µm, respectively).

Higher shear force was observed in line C (4.04 kgf) compared to lines A and B (3.68 kgf and 3.66 kgf, respectively), and higher sarcomere length and lower shear force were registered in castrated males (1.96 µm and 3.68 kgf, respectively) compared to females (1.94 µm and 3.90 kgf, respectively) (Table 2).

There was a statistical difference in marbling score between sexes (Table 2), with castrated males exhibiting higher marbling scores (2.74) than females (2.48). However, no significant difference in fat content was found between sexes (Table 3).

Among genetic groups, marbling score also differed statistically. Line C had higher marbling scores (2.82) compared to line A (2.43). Nevertheless, despite the higher marbling score in line C, no significant difference was observed in fat content among lines (Table 3). No significant differences were observed among genetic groups, sex, or their interaction for pH, lightness (L*), redness (a*), yellowness (b*), water-holding capacity (WHC), and cooking loss (CL).

The chemical composition and collagen content of pork among genetic groups, sex, and their interaction are shown in Table 3. A significant interaction (*p* < 0.05) was found between sex and genetic group for total, soluble, and insoluble collagen. Males from lines A and B showed higher insoluble, soluble and total collagen (Line A: 0.35%; 0.09%; 0.44% and Line B: 0.38%; 0.09%; 0.47%, respectively) than females of the correspondent line (Line A: 0.17%; 0.05%; 0.22% and Line B: 0.21%; 0.05%; 0.26%, respectively). Males of line C had lower insoluble, soluble and total collagen (0.18%; 0.04% and 0.21%, respectively) than those of lines A and B (0.35–0.38%; 0.09–0.09% and 0.44–0.47%, respectively).

Significant sex differences were found for protein and mineral content. Castrated males had higher protein and lower mineral content (24.73%, 1.55%, respectively) compared to females (23.91%, 1.85%, respectively). Among genetic groups, mineral content differed significantly. Line A had higher mineral content (1.90%) compared to lines B and C (1.60% each).

Regarding the scores, all variables evaluated in the sensory panel ranged between 6 and 7 (Table 4).

These scores indicated that the panelists slightly to moderately liked the meat samples. No statistical differences or interactions were observed for any of the evaluated variables.

## 4. Discussion

It is well established that sex plays an important role in carcass traits [2] and is one of the main factors influencing the increase in lean meat proportion [26]. Animals of different sexes exhibit distinct growth curves and body composition [27]. Castrated males grow and reach physiological maturity at a faster rate than females [28]. The absence of testicular hormones, such as testosterone—which has a key anabolic function—reduces protein synthesis, resulting in carcasses with higher fat content [29]. These characteristics lead to lower cut yields in castrated males compared to females [28,30].

Females demonstrate greater feed efficiency [31] and protein deposition than castrated males [10,11], which may explain the results observed in this study. Previous research has also reported higher ham yield in females compared to surgically castrated males [32]. Ham is the primary raw material for the production of processed ham, an economically important pork product [12]. Therefore, it is crucial to consider sex-specific carcass traits to determine the optimal processing method for ham [32].

When comparing carcass traits among commercial pigs, results depend on the specific breeds crossed [33]. Each breed and crossbreed exhibits considerable heterogeneity in traits [34]. Thus, differences between lines B and C may reflect variation in body structure of the breeds composing each line. Line B is composed of line A × Pietrain, while line C is line A × Hampshire. Breed composition, alongside carcass weight and sex, significantly affects the weight and proportion of most primal cuts [35]. Pietrain pigs are leaner and have higher overall cut yields compared to breeds similar to Hampshire (Duroc breed), and higher ham yield in Pietrain lines compared to other breeds has been documented [36]. This suggests that higher ham yield is a characteristic feature of the Pietrain breed, likely explaining the greater ham yield in line B compared to line C animals, despite the latter having higher hot and cold carcass weights.

Recognizing that different breeds have predetermined traits in carcass composition and meat quality parameters [4], and that pork cuts are key in economic valorization within the pork production chain [35], it is essential to consider the effect of genetic lines on weight and proportion of pork cuts, especially those processed into high-value products such as ham. On the other hand, the present study found limited effects of genetic line on carcass traits. Hampshire is generally considered genetically similar to the Duroc breed [33], which exhibits higher backfat thickness and carcass fat content [37,38]. Thus, animals from line C were expected to show greater backfat thickness and lower lean meat percentage compared to line B, which includes Pietrain known for producing lean meat [39]. One possible explanation is that lines B and C share a common genetic base, so the use of crossbred rather than purebred pigs might mask distinct carcass and quality traits characteristic of each sire line [36]. Another explanation is that breeding goals have simultaneously improved lean growth in Hampshire and fat quality in Pietrain. Generally, few differences are observed in commercial pigs for carcass traits, as pork production has long focused on similar targets: rapid growth and lean carcasses [33].

It is understood that sex influenced the genetic group. Several studies have reported differences in meat quality between surgically castrated males and female pigs. Castration is known to cause significant differences in intramuscular fat and fatty acid profiles [40]. These variations may be related to proteomic changes in the meat. A previous study reported that castration of male and female pigs induced major proteomic alterations in cured ham [41]. The authors suggested that proteomic differences in ham mainly reflected differences in the proteome of fresh meat. According to this study, structural proteins composing sarcomeres were differentially altered by castration, suggesting that the calpain system may lead to differential muscle degradation during early post-mortem, with a greater effect in castrated animals. Calpains are calcium-dependent cysteine proteases, existing mainly in two forms: μ-calpain and m-calpain. Among them, μ-calpain is one of the main enzymes responsible for post-mortem myofibrillar fragmentation [42]. Thus, the authors concluded that castration strongly influences structural protein degradation [41], which may explain the greater sarcomere length observed in surgically castrated males of line C compared to females of the same line. In this context, the greatest sex effect was observed in line C (Line A × Hampshire). Line C contains genetic material from Hampshire, which generally presents a lower ultimate meat pH compared to many other breeds [33,34].

Meat pH is one of the factors influencing μ-calpain activation [43]. A faster pH decline triggers earlier activation of μ-calpain, resulting in an earlier loss of its proteolytic activity. Slightly lower pH values may stimulate faster μ-calpain activation [44], potentially causing lower rates of myofibrillar fragmentation due to early enzyme inactivation. Although no statistical difference in ultimate pH was found among lines, line C animals may tend to have a faster post-mortem pH decline and, consequently, lower calpain activity. Therefore, calpain activity in castrated males of line C may have been more pronounced than in females of the same line, explaining the sarcomere length differences found. These differences may also explain the higher shear force observed in line C compared to lines A and B, and the higher sarcomere length and lower shear force in castrated males compared to females. Sarcomere structure also affects meat quality [45]. It is well established that increased sarcomere length favors reduced shear force [46] and improves meat tenderness [47], supporting the present findings.

Growth curves and body composition differ asymmetrically between castrated males and females. Castrated males grow and reach physiological maturity faster than females [28]. Thus, at the onset of fat deposition in castrated males, females are still growing and developing [35]. Consequently, castrated males have carcasses with higher fat content [28], consistent with the greater backfat thickness and lower lean meat percentage found in castrated males in this study.

Regarding marbling, Line C exhibited higher scores, which is consistent with its genetic background. Because Line C was developed through crossing with Hampshire, increased marbling and intramuscular fat deposition were expected. Hampshire and Hampshire-derived crossbreds are well documented for their tendency to accumulate greater intramuscular fat compared with other commercial breeds [33,48,49].

Numerous studies have reported differences in meat quality between castrated males and females, primarily due to their distinct growth curves and body composition [27]. However, findings regarding collagen content are inconsistent. Some studies in pigs and cattle found no effect of sex on soluble or total collagen [50,51], whereas other bovine studies reported that intact males exhibit increased collagen synthesis near puberty compared to castrates, potentially leading to greater insoluble collagen through additional cross-linking [52,53,54]. Notably, there is a lack of studies specifically addressing sex-related differences in pork collagen content [50,55]. Breed may also influence these differences, as breeds with higher collagen content at birth are more affected by castration than those with lower initial collagen [56]. In this context, our results suggest that Lines A and B synthesize more collagen than Line C, which may explain the higher total, soluble, and insoluble collagen observed in castrated males from these lines. These findings highlight the combined influence of sex and genetic background on collagen deposition in pork.

Genetic diversity among breeds also results in variations in meat quality [57]. Although all animals share a common genetic line, the crosses performed introduced meat quality variations, with differences in collagen synthesis among lines. Line A is a synthetic purebred genetic line selected for maximum profitability, growth efficiency, and carcass value. Line B (Line A × Pietrain) combines carcass conformation, growth efficiency, and robustness, while Line C (Line A × Hampshire) is characterized by high growth performance and intramuscular fat deposition. Early-maturing breeds typically show higher total and insoluble collagen, whereas fast-growing, later-maturing animals present higher soluble collagen [54,58]. In this study, Lines A and B exhibited higher total and insoluble collagen compared to Line C, suggesting that their genetic backgrounds favor earlier collagen deposition and structural protein development. Line C, influenced by Hampshire genetics, showed lower collagen content, which may be associated with later collagen maturation and greater emphasis on fat rather than connective tissue accumulation. These differences align with the expected physiological and carcass traits of each genetic line.

Regarding meat protein content, previous studies reported conflicting results between castrated males and females. Some found no statistical difference [59], while others reported higher protein in females [13,31,60,61], associating lower protein in males with higher intramuscular fat deposition. This was not observed here, as castrated males showed higher protein content, with no sex difference in fat content. Supporting the current findings, Kwasiborski et al. [62] reported higher protein concentration in castrated males, based on a proteomic analysis of the Longissimus lumborum muscle, attributing increased extractability of myofibrillar proteins (actin and myosin light chain isoforms) post-mortem in castrates. Actin and myosin form the actomyosin complex, characterized by strong cross-bridges [63]. Under the ionic strength used for protein extraction, actomyosin complexes are poorly soluble, limiting extraction compared to uncomplexed actin and myosin. The authors suggested extracted actin and myosin levels inversely relate to actomyosin complex amount, indicating fewer complexes in castrated males, possibly explaining higher protein content.

In study conducted by Gordon et al. [64], castrated males showed lower post-mortem intracellular Ca^2+^ levels, which are critical for actomyosin complex formation. Intracellular Ca^2+^ levels increase in the presence of estradiol and testosterone [65,66]; thus, the absence of testosterone in castrates may explain their lower Ca^2+^ levels compared to females, potentially already present in live animals. These differences may contribute to variations in pork quality, such as protein content, linked to proteomic muscle changes [41]. However, protein quantification in the present study differs from that reported previously, and further research is recommended to clarify these observations.

Lower mineral content in castrated males compared to females was also reported previously [67]. Sex influences meat quality parameters due to differences in protein and fat deposition among sexes [51]. Muscle development and fat deposition are important for chemical meat quality [68]. Mineral content in lean meat inversely correlates with fat content [69]. Castrated males typically have carcasses with higher fat content [29], consistent with greater backfat and lower lean meat percentages found here. Although no significant sex difference was found for fat content, castrated males had higher fat values (3.23%). Differences in growth curves and body composition between castrated males and females [27] likely caused mineral content variations between sexes. Although Line C presented a higher visual marbling score, this difference was not accompanied by higher chemically determined intramuscular fat. This apparent discrepancy is consistent with previous reports showing that marbling score reflects not only the amount but also the distribution of visible fat within the muscle. Hampshire-influenced lines, such as Line C, typically exhibit coarser and more conspicuous intramuscular fat deposits, which increase visual marbling even when total lipid content is statistically similar among groups. Therefore, the higher marbling score observed in Line C likely reflects breed-specific fat distribution patterns rather than greater absolute fat deposition.

Lines B and C were developed by crossing line A with Pietrain and Hampshire genetic lines, suggesting crosses influenced mineral content, resulting in lower values in lines B and C. Crossbreeding promotes trait complementarity and maximizes heterosis [70]. The industry should consider market demands and develop new breeding strategies or programs to ensure production of pork with desired characteristics [71].

Regarding sensory analysis, most panelists did not perceive differences among samples, indicating that consumers were generally unable to distinguish pork from the three genetic lines under the tested conditions. This is a positive outcome, since mean scores (6–7) reflect good acceptance for appearance, aroma, flavor, texture and overall liking. Several factors likely explain the absence of sensory discrimination: samples were prepared under standardized cooking conditions that tend to minimize perceptible variability; the panel consisted of untrained consumers, whose detection thresholds for subtle differences in tenderness, juiciness and flavor are higher than those of trained assessors; and the instrumental differences observed, although statistically significant, were probably below the sensory detection threshold. Importantly, this lack of sensory differentiation does not imply that physicochemical traits are irrelevant to eating quality, these traits remain biologically and technologically important, but it indicates that the specific consumer panel and protocol used here were not sensitive enough to capture small differences. Finally, the results suggest that, within contemporary high-performance commercial lines and under typical preparation conditions, pork quality is maintained at a level that meets consumer expectations.

## 5. Conclusions

Sex and genetics influence carcass traits and pork quality, reflecting the inherent biological and genetic potential of new commercial lineages. Castrated males showed greater backfat, higher marbling and tenderness, with longer sarcomeres, higher protein content, lower lean meat percentage and mineral content than females, indicating sex-specific effects on muscle composition and meat structure. Line C exhibited the highest carcass weights, marbling scores and sarcomere length, consistent with Hampshire influence. Line B achieved the highest ham yield, reflecting Pietrain-related carcass conformation, and Lines A and B higher collagen synthesis than Line C, suggesting differences in growth and maturation patterns. However, sensory perception is not always sensitive enough to detect differences in pork from different sexes and genetic lines, even when instrumental analyses reveal variations. Therefore, untrained consumers perceived the pork as similar, and all samples met expectations for appearance, flavor, texture and overall liking.

## Figures and Tables

**Table 1:** Hot carcass weight (HCW), cold carcass weight (CCW), muscle depth (MD), backfat thickness (BT), lean meat percentage (LMP), and yield of loin, ham, rack and shoulder cuts from pigs of different sexes and genetic groups.

Variables	Sex		Genetic Group (GG)			SEM	*p*-Value		
	Male	Female	Line A	Line B	Line C		Sex	GG	Sex × GG
HCW (kg)	88.64	87.29	88.60 ab	85.25 b	90.04 a	1.1407	0.2981	0.0089	0.4090
CCW (kg)	85.99	85.22	86.05 ab	82.67 b	88.05 a	1.1082	0.5366	0.0022	0.2277
MD (mm)	73.01	73.33	73.39	72.40	73.70	0.6663	0.6746	0.3440	0.1112
BT (mm)	14.05 a	13.38 b	13.55	13.61	13.90	0.1597	0.0031	0.2287	0.6397
LMP (%)	58.65 b	59.08 a	59.01	58.84	58.75	0.1461	0.0397	0.5840	0.2609
Loin (%)	8.29	8.17	8.24	8.28	8.17	0.1152	0.3405	0.7678	0.5643
Ham (%)	31.88 b	32.29 a	32.12 ab	32.50 a	31.64 b	0.1709	0.0368	0.0021	0.2504
Rack (%)	17.76	17.53	17.34	17.57	17.62	0.1365	0.1440	0.6616	0.5515
Shoulder (%)	21.99	21.88	21.78	21.97	22.07	0.1227	0.4279	0.2287	0.6420

^a,b^ Means followed by different letters in the rows differ significantly according to Tukey’s test (*p* < 0.05). SEM = Standard error of the mean.

**Table 2:** pH values, lightness (L*), redness (a*), yellowness (b*), marbling score, water-holding capacity (WHC), cooking loss (CL), shear force (SF) and interaction for sarcomere length (SL) of pork from different sexes and genetic groups.

**Variables**	**Sex**		**Genetic Group (GG)**			**SEM**	***p*-Value**		
	**Male**	**Female**	**Line A**	**Line B**	**Line C**		**Sex**	**GG**	**Sex × GG**
pH	5.57	5.53	5.53	5.54	5.57	0.0189	0.0986	0.3287	0.9024
L*	55.10	55.35	55.39	55.62	54.66	0.5463	0.6965	0.4245	0.5646
a*	9.25	8.69	9.36	8.69	8.88	0.2947	0.0921	0.2483	0.7123
b*	2.17	2.15	2.27	2.42	1.79	0.2086	0.9458	0.0800	0.6988
Marbling	2.74 a	2.48 b	2.43 b	2.57 ab	2.82 a	0.1111	0.0403	0.0409	0.4924
WHC (%)	75.65	76.15	75.81	76.25	75.56	0.5863	0.4219	0.7001	0.0945
CL (%)	26.68	28.25	26.84	27.25	28.30	0.9014	0.1269	0.4944	0.9906
SF (kgf)	3.68 b	3.90 a	3.68 b	3.66 b	4.04 a	0.9556	0.0402	0.0061	0.1193
	**Sex × Genetic Group**								
	**Line A**		**Line B**		**Line C**		**SEM**	***p*** **-Value**	
	**Male**	**Female**	**Male**	**Female**	**Male**	**Female**			
SL (µm)	1.95 AB	1.95 AB	1.94 B	1.95 AB	1.99 Aa	1.92 Bb	0.0070	0.0002	

Means of marbling, shear force (SF) and sarcomere length (SL) followed by different letters in the rows differ significantly (Tukey’s test, *p* < 0.05). For SL, lowercase letters indicate differences between sexes within a genetic group, while uppercase letters indicate differences between sexes across genetic groups. SEM = Standard Error of the Mean.

**Table 3:** Values of protein, fat, moisture, mineral matter (MM), and interaction between sex and genetic group for total, soluble and insoluble collagen in the pork.

**Variables**	**Sex**		**Genetic Group (GG)**			**SEM**	***p*-Value**		
	**Male**	**Female**	**Line A**	**Line B**	**Line C**		**Sex**	**GG**	**Sex × GG**
Protein (%)	24.73 a	23.91 b	24.41	24.28	24.28	0.2749	0.0379	0.9517	0.8956
Fat (%)	3.23	3.11	3.32	3.05	3.14	0.1536	0.4827	0.4483	0.4703
Moisture (%)	70.32	70.24	69.89	70.37	70.58	0.2696	0.7905	0.1711	0.3336
MM (%)	1.55 b	1.85 a	1.90 a	1.60 b	1.60 b	0.0745	0.0007	0.0039	0.1410
**Sex**	**Insoluble Collagen (%)**					***p*** **-Value**		**SEM**	
	**Line A**		**Line B**		**Line C**				
Male	0.35 Aa		0.38 Aa		0.18 b	<0.0001		0.0166	
Female	0.17 B		0.21 B		0.16				
**Sex**	**Soluble Collagen (%)**					***p*** **-Value**		**SEM**	
	**Line A**		**Line B**		**Line C**				
Male	0.09 Aa		0.09 Aa		0.04 b	0.0262		0.0037	
Female	0.05 B		0.05 B		0.03				
**Sex**	**Total Collagen (%)**					***p*** **-Value**		**SEM**	
	**Line A**		**Line B**		**Line C**				
Male	0.44 Aa		0.47 Aa		0.21 b	<0.0001		0.0187	
Female	0.22 B		0.26 B		0.20				

Means of protein, mineral matter (MM) and collagen (total, soluble and insoluble) followed by different letters in the rows differ significantly (Tukey’s test, *p* < 0.05). For collagen, lowercase letters indicate differences across genetic groups, while uppercase letters indicate differences between sex. SEM = Standard Error of the Mean.

**Table 4:** Sensory analysis of pork from different sexes and genetic groups.

Variables	Sex		Genetic Group (GG)			SEM	*p*-Value		
	Male	Female	Line A	Line B	Line C		Sex	GG	Sex × GG
Appearance	6.47	6.52	6.46	6.58	6.45	0.1566	0.6958	0.6425	0.2170
Aroma	6.83	6.78	6.70	6.93	6.79	0.1518	0.6672	0.3144	0.9821
Flavor	6.64	6.63	6.52	6.75	6.64	0.1670	0.9028	0.3879	0.8060
Texture	6.96	6.87	6.92	6.93	6.91	0.1542	0.4587	0.9868	0.8235
Acceptability	6.88	6.80	6.79	6.87	6.85	0.1449	0.4992	0.8478	0.4716

SEM = Standard error of the mean.

## Data Availability

The data that support the findings is available in Repositorio Institucional da UNESP at https://repositorio.unesp.br/entities/publication/e7770f9a-de4f-4e3c-ac4e-8db628dd272c, accessed on 14 December 2025.
